# Supplementary material for: Development and external validity of a nurse-led intervention program to improve palliative care and quality of dying and death in intensive care unit
Source: PLoS One. 2026 Apr 10;21(4):e0346585. doi: 10.1371/journal.pone.0346585 (PMC13068268; doi:10.1371/journal.pone.0346585)
Supplement: S1 Text — (DOCX) [file pone.0346585.s001.docx]

**S1 Text. Survey instrument used for content validity indexes evaluation**

1. **Care Recipients Under the Intervention Program**

Based on your feedback from the interview, we have revised the content regarding “Care Recipients Under the Intervention Program” Please review the content and let us know whether the revised content is appropriate.

| Evaluation Items | not valid | somewhat lacking validity | fairly valid | valid |
| --- | --- | --- | --- | --- |
| 1. The “Intervention Program Flow Diagram” has been structurally revised and reformatted into a ladder framework and renamed as the “Quality improvement ladder for palliative care and quality of dying and death in the ICU.” Please provide your comments on the revised content. Please provide your comments on the revised content. | 1 | 2 | 3 | 4 |
| Other feedback (free-text response)　[　　　　　　　　　　　　　　　　　　　　　　　　　　　　　　　　] | | | | |
| 1. The “Initial Screening Items” and “Basic Information on the Quality of Dying and Death” have been revised. Please provide your comments on the revised content. | 1 | 2 | 3 | 4 |
| Other feedback (free-text response)　[　　　　　　　　　　　　　　　　　　　　　　　　　　　　　　　　] | | | | |

1. **Provision of evidence-based symptom management**

Based on your feedback from the interview, we have revised the content regarding “Provision of evidence-based symptom management” Please review the content and let us know whether the revised content is appropriate.

| Evaluation Items | not valid | somewhat lacking validity | fairly valid | valid |
| --- | --- | --- | --- | --- |
| 1. Revisions have been made regarding the timing of the “Symptom Management and Palliative Care Conference.” Please provide your comments on these revisions. | 1 | 2 | 3 | 4 |
| Other feedback (free-text response)　[　　　　　　　　　　　　　　　　　　　　　　　　　　　　　　　　] | | | | |
| 1. Revisions have been made to the evaluation tool for “Provision of Evidence-Based Symptom Management.” Please provide your comments on these revisions. | 1 | 2 | 3 | 4 |
| Other feedback (free-text response)　[　　　　　　　　　　　　　　　　　　　　　　　　　　　　　　　　] | | | | |
| 1. Revisions have been made to the wording of “Provision of Evidence-Based Symptom Management.” Please provide your comments on these revisions. | 1 | 2 | 3 | 4 |
| Other feedback (free-text response)　[　　　　　　　　　　　　　　　　　　　　　　　　　　　　　　　　] | | | | |

1. **Multidisciplinary Bedside Conferences**

Based on your feedback from the interview, we have revised the content regarding “Multidisciplinary Bedside Conferences” Please review the content and let us know whether the revised content is appropriate.

| Evaluation Items | not valid | somewhat lacking validity | fairly valid | valid |
| --- | --- | --- | --- | --- |
| 1. Revisions have been made regarding the timing of the “Multidisciplinary Bedside Conference.” Please provide your comments on these revisions. | 1 | 2 | 3 | 4 |
| Other feedback (free-text response)　[　　　　　　　　　　　　　　　　　　　　　　　　　　　　　　　　] | | | | |
| 1. Revisions have been made to the conference items for the “Multidisciplinary Bedside Conference.” Please provide your comments on these revisions. | 1 | 2 | 3 | 4 |
| Other feedback (free-text response)　[　　　　　　　　　　　　　　　　　　　　　　　　　　　　　　　　] | | | | |

1. **Family Conference**

Based on your feedback from the interview, we have revised the content regarding “Family Conference” Please review the content and let us know whether the revised content is appropriate.

| Evaluation Items | not valid | somewhat lacking validity | fairly valid | valid |
| --- | --- | --- | --- | --- |
| 1. Revisions have been made to the procedures of the “Family Conference” (including timing, participants, and process). Please provide your comments on these revisions. | 1 | 2 | 3 | 4 |
| Other feedback (free-text response)　[　　　　　　　　　　　　　　　　　　　　　　　　　　　　　　　　] | | | | |
| 1. Revisions have been made to the wording of the “Family Conference.” Please provide your comments on these revisions. | 1 | 2 | 3 | 4 |
| Other feedback (free-text response)　[　　　　　　　　　　　　　　　　　　　　　　　　　　　　　　　　] | | | | |

1. **End-of-Life Care**

Based on your feedback from the interview, we have revised the content regarding “End-of-Life Care” Please review the content and let us know whether the revised content is appropriate.

| Evaluation Items | not valid | somewhat lacking validity | fairly valid | valid |
| --- | --- | --- | --- | --- |
| 1. Revisions have been made to the procedures of “End-of-Life Care” (including timing and process). Please provide your comments on these revisions. | 1 | 2 | 3 | 4 |
| Other feedback (free-text response)　[　　　　　　　　　　　　　　　　　　　　　　　　　　　　　　　　] | | | | |
| 1. Revisions have been made to the content of “End-of-Life Care.” Please provide your comments on these revisions. | 1 | 2 | 3 | 4 |
| Other feedback (free-text response)　[　　　　　　　　　　　　　　　　　　　　　　　　　　　　　　　　] | | | | |
