# Supplementary material for: Development and external validity of a nurse-led intervention program to improve palliative care and quality of dying and death in intensive care unit
Source: PLoS One. 2026 Apr 10;21(4):e0346585. doi: 10.1371/journal.pone.0346585 (PMC13068268; doi:10.1371/journal.pone.0346585)
Supplement: S2 Text — (DOCX) [file pone.0346585.s002.docx]

**S2 Text. Item-level content validity indexes ratings**

| **Program components** | Expert1 | Expert2 | Expert3 | Expert4 | Expert5 | Expert6 | I-CVI |
| --- | --- | --- | --- | --- | --- | --- | --- |
| Evaluation Items (Revision items) |  |  |  |  |  |  |  |
| **Care Recipients Under the Intervention Program** | | | | | | | |
| 1. The program content was restructured into a tiered forma | 4 | 3 | 4 | 4 | 4 | 2 | 0.83 |
| 1. Revised on “Initial screening items” and “Basic Information Related to the Quality of End-of-Life Care.” | 3 | 4 | 4 | 4 | 4 | 2 | 0.83 |
| **Provision of evidence-based symptom management** | | | | | | | |
| 1. Methods (Timing, Participants, Procedures, etc.) | 3 | 4 | 4 | 4 | 3 | 3 | 1.00 |
| 1. Use of Japanese Evidence | 3 | 4 | 4 | 4 | 4 | 3 | 1.00 |
| 1. Revisions to Wording | 3 | 4 | 4 | 4 | 4 | 3 | 1.00 |
| **Multidisciplinary Bedside Conferences** | | | | | | | |
| 1. Methods (Timing, Participants, Procedures, etc.) | 3 | 4 | 3 | 4 | 4 | 3 | 1.00 |
| 1. Use of Japanese Evidence | 3 | 4 | 4 | 4 | 4 | 2 | 0.83 |
| **Family Conference** | | | | | | | |
| 1. Methods (Timing, Participants, Procedures, etc.) | 3 | 3 | 3 | 4 | 4 | 3 | 1.00 |
| 1. Revisions to Wording | 3 | 3 | 4 | 4 | 2 | 3 | 0.83 |
| **End-of-Life Care** | | | | | | | |
| 1. Methods (Timing, Participants, Procedures, etc.) | 3 | 3 | 4 | 4 | 4 | 2 | 0.83 |
| 1. Use of Japanese Evidence | 3 | 3 | 3 | 4 | 4 | 3 | 1.00 |

I-CVI, Item-level content validity index
