# Supplementary material for: Development and external validity of a nurse-led intervention program to improve palliative care and quality of dying and death in intensive care unit
Source: PLoS One. 2026 Apr 10;21(4):e0346585. doi: 10.1371/journal.pone.0346585 (PMC13068268; doi:10.1371/journal.pone.0346585)
Supplement: S3 Text — (DOCX) [file pone.0346585.s003.docx]

**S3 Text. Nurse-Led Intervention Program to Improve the Palliative care and Quality of Dying and Death in Intensive Care Unit**


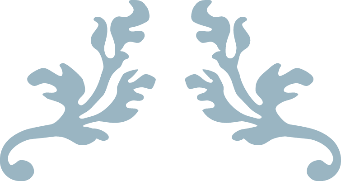


**Nurse-Led Intervention Program to Improve the**

**Palliative care and Quality of Dying and Death**

**in Intensive Care Unit**


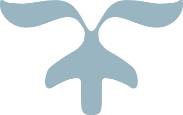


**CONTENTS**

[**Ⅰ. Objective** 2](#_Toc202940105)

[**Ⅱ. Overview** 2](#_Toc202940106)

[**Ⅲ. Contents** 2](#_Toc202940107)

[**Ladder Ⅰ: Provision of evidence-based symptom management** 2](#_Toc202940108)

[**1.** **Pain** 3](#_Toc202940109)

[**2.** **Dyspnea** 5](#_Toc202940110)

[**3.** **Agitation/delirium** 6](#_Toc202940111)

[**4.** **Thirst and dry mouth** 9](#_Toc202940112)

[**5.** **Sleep disturbance and anxiety** 10](#_Toc202940113)

[**6.** **Palliative sedation at the EOL** 11](#_Toc202940114)

[**Ladder Ⅱ: Multidisciplinary and family bedside conferences** 12](#_Toc202940115)

[**Family conferences** 12](#_Toc202940116)

[**1.** **Explanation to the patient and family** 13](#_Toc202940117)

[**2.** **Confirmation of patient values and wishes regarding current treatment options** 13](#_Toc202940118)

[**3.** **Determination of treatment and care goals** 13](#_Toc202940119)

[**4.** **Assessment of family needs** 13](#_Toc202940120)

[**Ladder Ⅲ: EOL care** 14](#_Toc202940121)

[**1.** **Symptom relief** 14](#_Toc202940122)

[**2.** **Provision of EOL information and confirmation of family understanding** 14](#_Toc202940123)

[**3.** **Identifying and fulfilling the needs of the patient and family in relation to the bereavement** 15](#_Toc202940124)

[**4.** **Psychosocial support** 16](#_Toc202940125)

[**5.** **Post-bereavement follow-up** 17](#_Toc202940126)

[**Figure 1.** Quality improvement ladder for palliative care and quality of dying and death in the ICU 18](#_Toc202940127)

[**Figure 2.** The four topics approach to clinical ethics case analysis 19](#_Toc202940128)

[**References** 20](#_Toc202940129)

[**Supplementary material:** Communication guide for patients and families 22](#_Toc202940130)

[**1. SPIKES (Setup / Perception / Invitation / Knowledge / Emotion / Summarize)** 22](#_Toc202940131)

[**2. NURSE (Naming/Understanding/Respecting/ Supporting/Exploring)** 23](#_Toc202940132)

[**3. REMAP (Reframe / Expect / Map / Align / Plan)** 24](#_Toc202940133)

# **Ⅰ. Objective**

This quality intervention program was designed to improve the quality of end-of-life (EOL) care and the quality of dying and death (QODD) in the intensive care unit (ICU). It involves repeated assessments and targeted interventions based on the ‘Quality Improvement Ladder for Palliative Care and Quality of Dying and Death in the ICU.’ Through this structured approach, the program was designed with the aim of supporting effective symptom management and shared decision-making from the early phase of ICU admission. Additionally, we aimed to facilitate timely transition to EOL care and provide comprehensive support throughout the dying process, thereby enhancing the overall quality of care provided to critically ill patients and their families.

# **Ⅱ. Overview**

The results of screening are used to determine the appropriate application of Ladders I, II, or III. Each ladder consists of evidence-based symptom management, multidisciplinary bedside conferences, family meetings, and EOL care (Figure 1).

# **Ⅲ. Contents**

## **Ladder Ⅰ: Provision of evidence-based symptom management**

Interventions are performed for patients who meet the primary screening criteria, and implementation is initiated after screening. Nurses conduct daily assessments in relation to pain, dyspnea, restlessness/delirium, dry mouth, and sleep disturbances/anxiety, and document their findings (Appendix 2). Additional symptoms, if present, are evaluated. Based on the assessment findings, a symptom management and relief conference is held daily to determine the need for additional symptom-focused interventions and to discuss appropriate strategies. This conference can be integrated into existing meetings and clinical rounds. Ladder I screening is conducted concurrently with the nurses during the conference. If the screening criteria are met, the patient proceeds to Ladder II.

Symptom assessment methods and standards of care are outlined in Sections (1) and (6) below. The standard of care is based on established guidelines and literature, including ‘Improving palliative care in the ICU’ (Puntillo et al., 2014); ‘Pain, agitation/sedation, delirium, immobility (rehabilitation/mobilization), and sleep guidelines’ (Devlin et al, 2018); ‘Comfort care’ (Blinderman et al., 2015); and the Japanese Society of Critical Care Nursing’s 2021 ‘Practical guide for oral care of intubated patients.’

### **Pain**

**Assessment method**

- Pain is assessed daily, minimally every 8 h. Additional assessments should be conducted when analgesic dosages are modified or when supplemental doses are administered.
- If a patient is able to self-report, pain should be assessed using a numeric rating scale (NRS) based on the results of subjective evaluation.


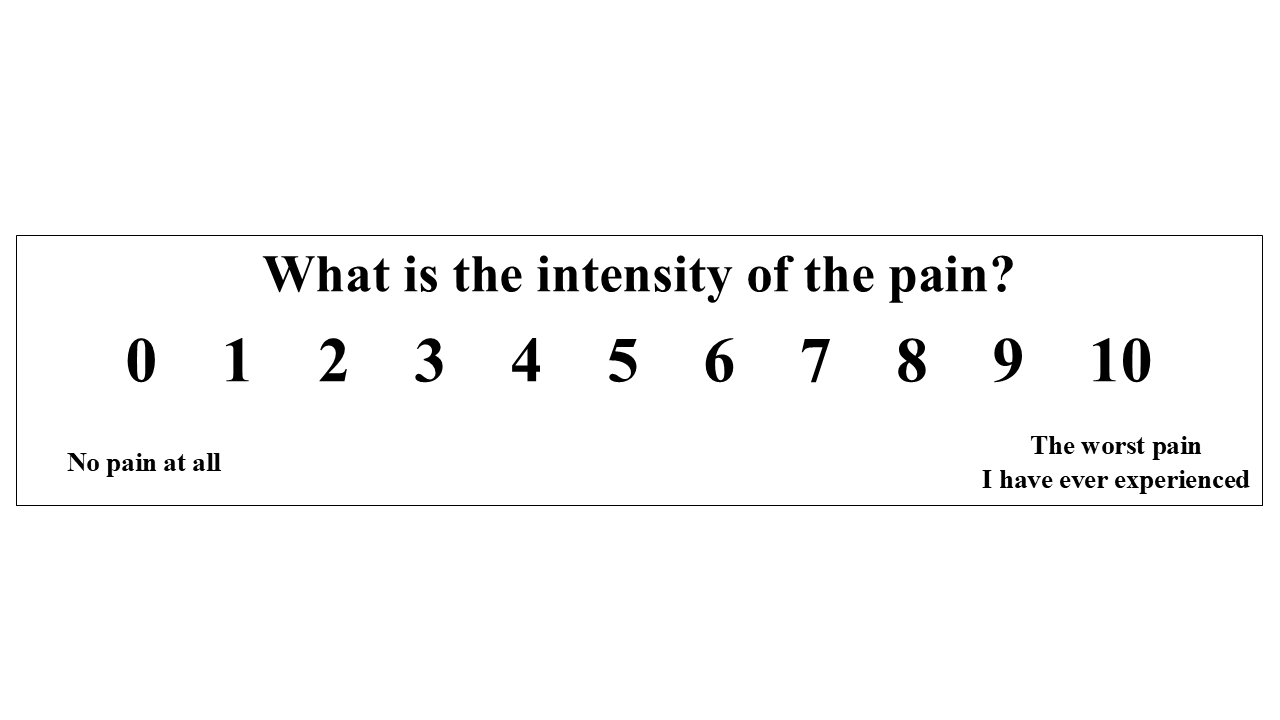


- If self-reporting is not feasible, pain should be assessed using objective tools employed within the unit, such as the Japanese version of the Behavioral Pain Scale (Payen et al., 2001) or the Japanese version of the Critical Care Pain Observation Tool (Yamada et al., 2016).


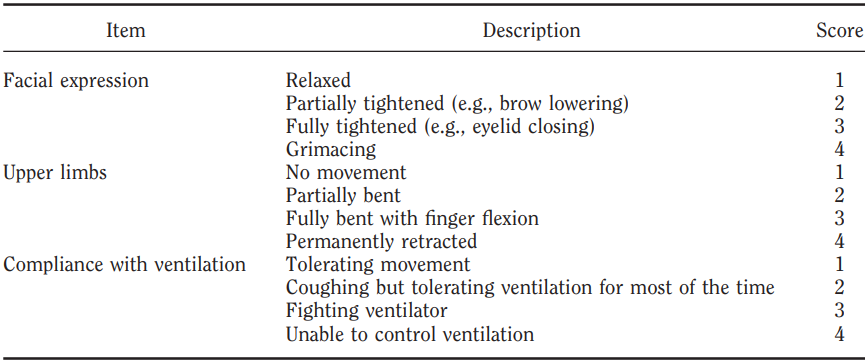


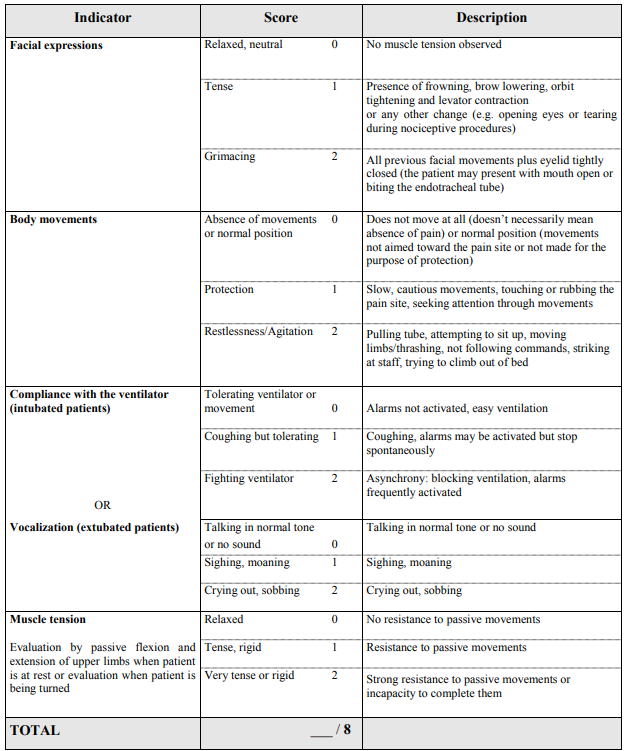


**Standard of care based on assessment results**

- If pain control is inadequate, the attending nurse may suggest the use of opioids as first-line treatment to the attending physician.
- If the analgesic effect of opioids is insufficient, the attending nurse may recommend the addition of other agents such as acetaminophen or NSAIDs.
- When pain management is challenging, the attending nurse may propose the use of low-dose ketamine to the attending physician (0.5 mg/kg IV bolus once, followed by a continuous infusion of 1–2 µg/kg/min).
- Combining opioids with adjuvant medications for neuropathic pain (e.g., gabapentin or carbamazepine) may also be suggested.
- The attending nurse and physician should discuss the need for an opioid bolus prior to procedures such as suctioning, repositioning, or drain removal.
- Non-pharmacological interventions, such as positioning, massage (limited to the back, limbs, or hands), and music therapy, may also be considered.
- The effectiveness of pain management interventions should be regularly evaluated and documented in the patient’s medical records.

### **Dyspnea**

**Assessment method**

- Evaluations are performed daily at least every 8 h. Assessments should also be conducted when ventilator settings are adjusted or when interventions for dyspnea are implemented.
- If the patient can self-report, dyspnea should be assessed using the NRS based on subjective evaluation findings.


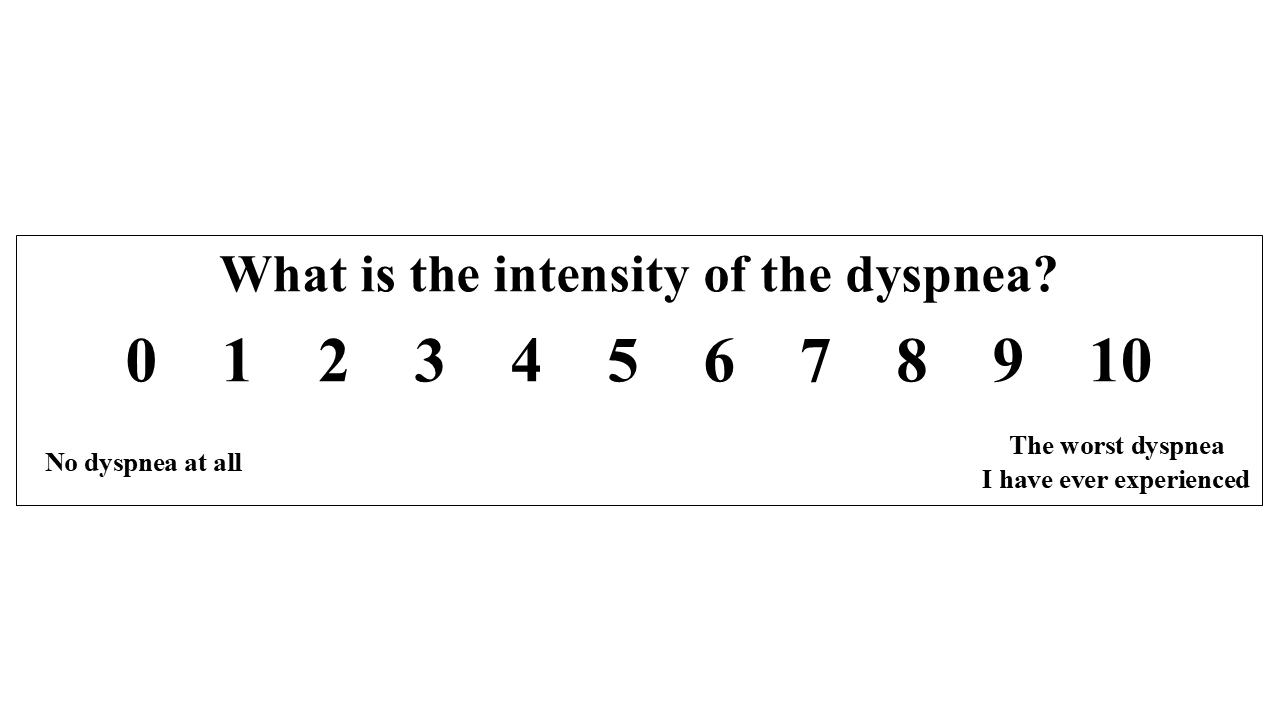


- If self-reporting is difficult, dyspnea should be assessed using an objective assessment tool already implemented in the department, or the Japanese version of the Objective Dyspnea Rating Scale (Sakuramoto et al., 2021). A score ≥3 indicates the presence of dyspnea, with higher scores indicating greater severity.


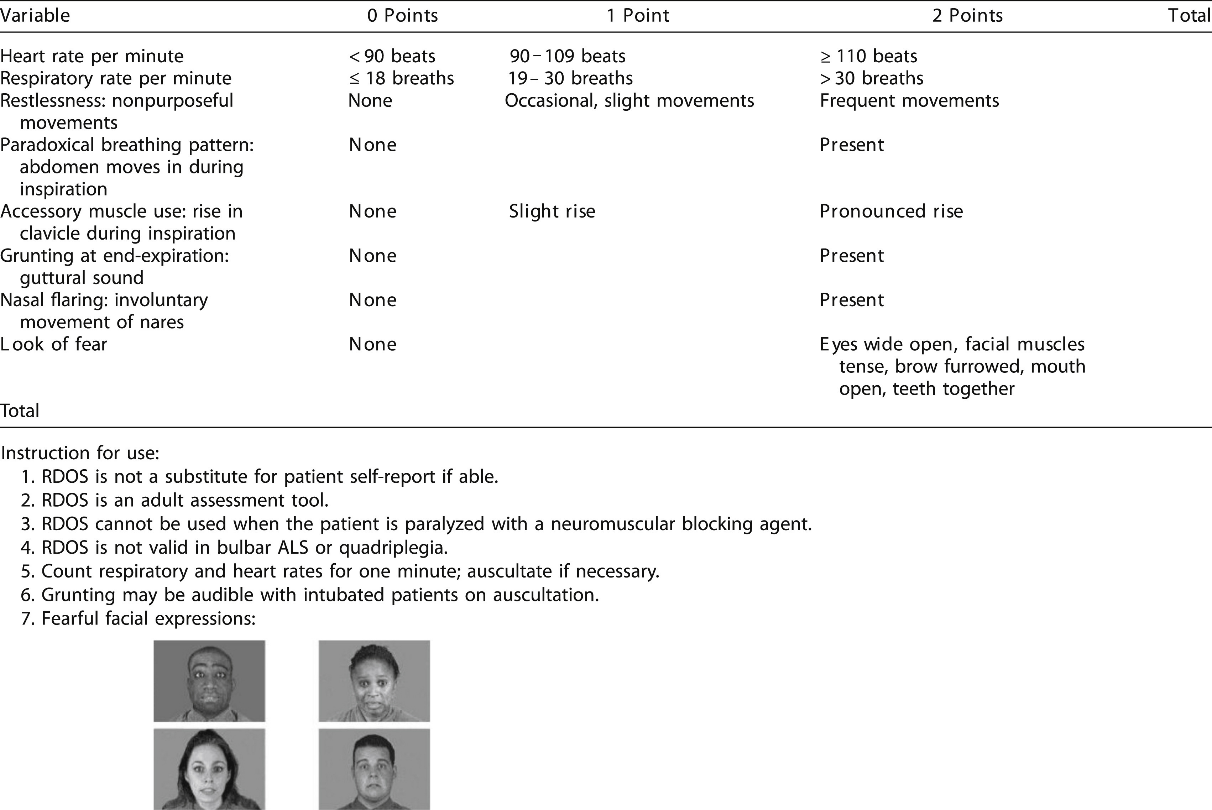


**Standard of care components based on assessment results**

- Optimize positioning to increase tidal volume
- Balance activity and rest to reduce excessive oxygen consumption
- In discussion with the attending physician, document the physician's instructions and apply the appropriate oxygen flow rate
- Consider implementing relaxation techniques (e.g., progressive muscle relaxation)
- Apply a fan or cool cloth to the face to stimulate the trigeminal nerve
- Recommend the use of opioids to the attending physician
- Suggest the adjunctive use of benzodiazepines in addition to the opioid regimen to the attending physician
- Evaluate the effectiveness of the interventions for dyspnea and document the findings in the medical records

### **Agitation/delirium**

**Assessment method**

- Conduct assessments at least every 8 h each day
- Assess for agitation and sedation depth using the Richmond Agitation–Sedation Scale (RASS) as an objective assessment tool to evaluate whether the sedation target set for treatment has been maintained. If no sedation target has been established, this will need to be determined at a clinical conference.
- Use Koga et al.’s (2014) Confusion Assessment Method for the Intensive Care Unit or the Intensive Care Delirium Checklist (Bergeron, 2001) to assess the patient’s delirium status.


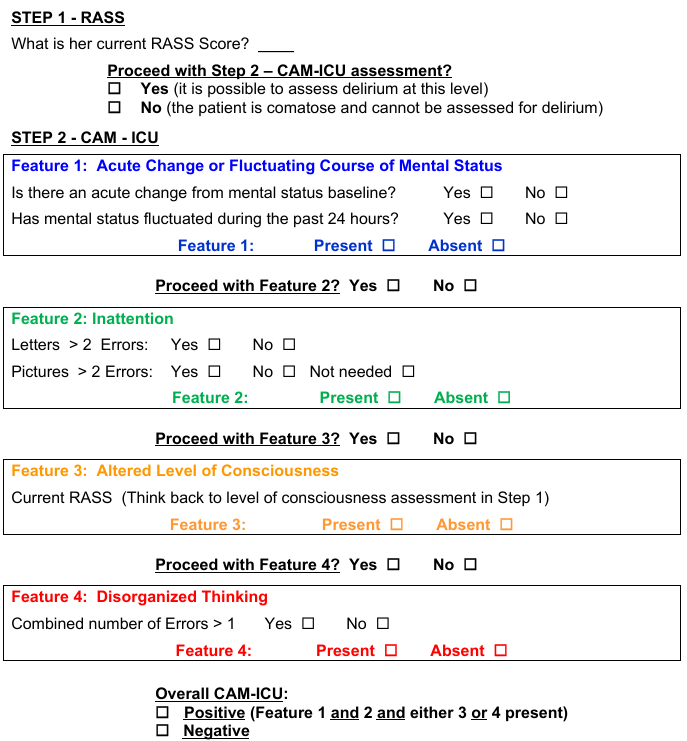


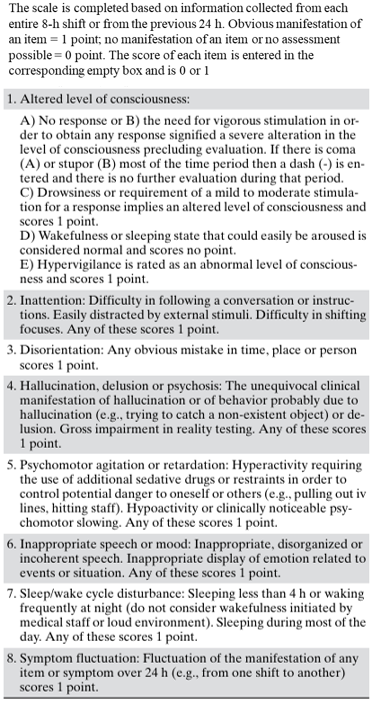


**Standard of care components based on assessment results**

- Set goals for an appropriate depth of sedation

Light sedation (RASS ≥-2) is recommended for early weaning from mechanical ventilation; however, an EOL optimal depth of sedation is not yet clearly established. Therefore, healthcare providers should reach a consensus through conferences and similar discussions.

- Nurses should titrate sedation within the range prescribed by the physicians, aiming to achieve the targeted level of sedation.
- Benzodiazepines may be used as adjuncts to relieve dyspnea; however, their routine use is discouraged because of the risk of delirium.
- At least every 8 h, consider whether physical restraints should be initiated, continued, or discontinued, preferably through a multidisciplinary discussion.
- Reduce modifiable risk factors for delirium through multifaceted non-pharmacological interventions such as cognitive stimulation (e.g., use of clocks and calendars, music), environmental modifications (e.g., minimizing light and noise), and addressing sensory impairments (e.g., use of hearing aids or glasses).
- Evaluate and document in the medical records the effectiveness of interventions aimed at preventing or alleviating agitation and delirium.

### **Thirst and dry mouth**

**Assessment method**

- Perform evaluations at least every 8 h daily
- If a patient can self-report, assess thirst and dry mouth using the NRS based on the results of subjective evaluation


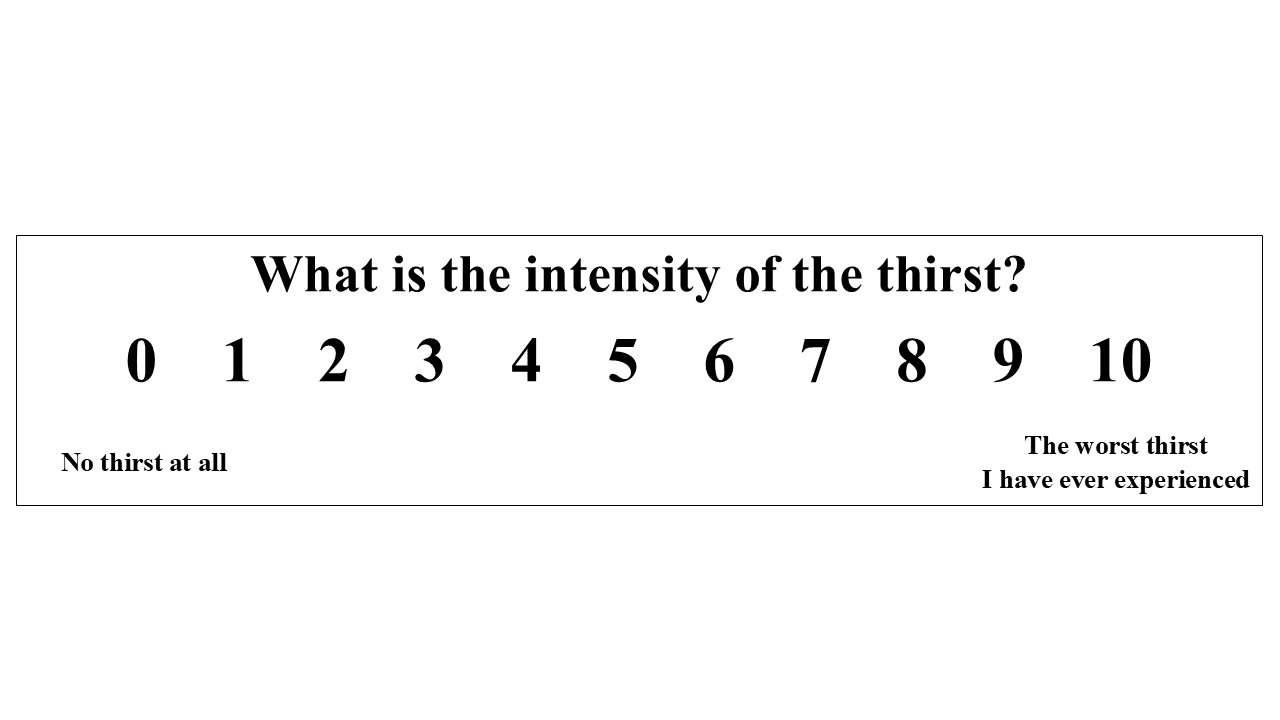


- If self-reporting is difficult, assess oral dryness using objective available departmental assessment tools or clinical diagnostic criteria for xerostomia such as the Oral Assessment Guide or the Revised Oral Assessment Guide (Kakinoki et al., 2008).


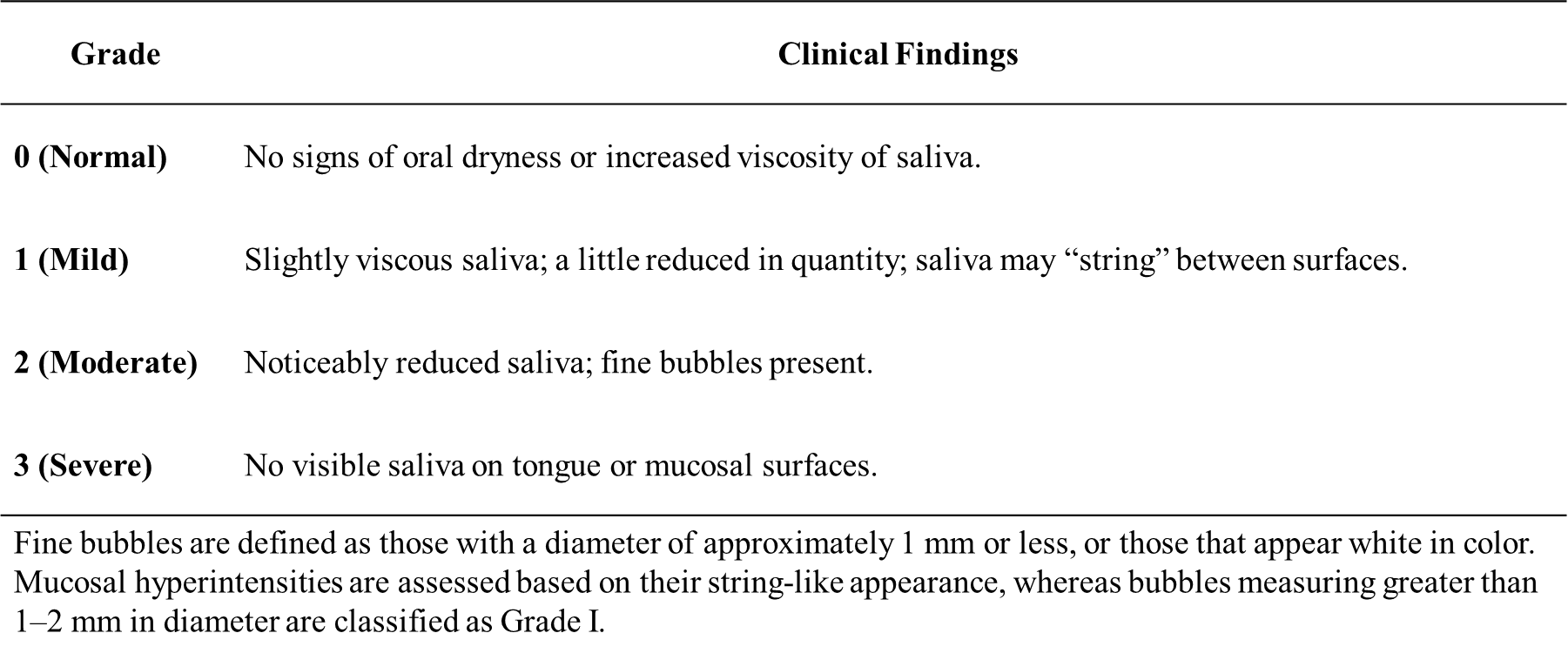


**Standard of care components based on assessment results**

- Provide frequent oral care
- Regularly apply water-soaked gauze, water spray, or ice chips to maintain oral moisture
- Consider the use of artificial saliva or moisturizing gel
- Evaluate the effectiveness of interventions for dry mouth and document the findings in the medical records
- If a patient's mouth stays open, cover the mouth with a mask or other protective measures to prevent dryness

### **Sleep disturbance and anxiety**

**Assessment method**

- Conduct daily assessments during daytime shifts
- If a patient is capable of self-reporting, assess sleep quality using the Richard-Campbell Sleep Questionnaire (Murata, 2019)
- Assess for the presence of anxiety through communication with the patient and, if possible, investigate the underlying causes


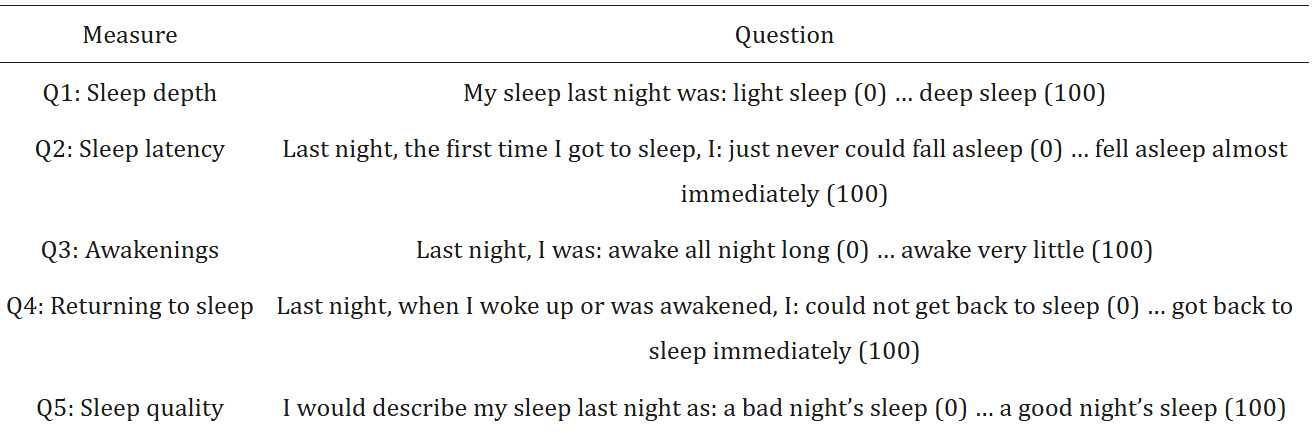


**Standard of care components based on assessment results**

- Propose to the attending physician a change in ventilator settings during the night (e.g., switching from pressure support ventilation (PSV) to assisted cycles (A/C) mode at night only)
- When requested by a patient, supply earplugs or eye masks to reduce noise and light
- Incorporate music into the care plan if desired by a patient
- Consider adjusting the night-time dosage of sedatives within the scope of the physician’s orders
- Anxiety is often associated with symptoms such as dyspnea, in which case a review of symptom assessment and standard care practices should be undertaken
- Listen attentively to patient concerns and determine causes of anxiety, and consider appropriate interventions through a multidisciplinary team approach
- Evaluate the effectiveness of interventions for sleep disturbance or anxiety, and document the outcomes in the medical records

### **Palliative sedation at the EOL**

Palliative sedation, which renders a patient unable to communicate, should only be considered as a last resort when refractory distressing symptoms cannot be controlled using any other means. In such cases, it is essential that the patient or their surrogate decision-maker provides informed consent and recognizes it as a legitimate approach.

Prior to initiating palliative sedation, a multidisciplinary bedside conference should be held, followed by a family meeting to ensure a thorough discussion with the patient and/or family. If a surrogate decision-maker is unavailable or unable to decide, consultation with palliative care specialists, psychiatrists, or an ethics committee should be considered.

## **Ladder Ⅱ: Multidisciplinary and family bedside conferences**

- Purpose: To establish a shared understanding among healthcare professionals regarding patient treatment and care goals
- Timing: Conducted for patients who meet the Ladder I screening criteria

The initial conference should be held within 3 days of the screening decision, and subsequent conferences should be held every 3 days. They may also be incorporated into existing conferences and rounds.

- Participants: Physician and nurse participation is mandatory, whereas there is flexibility in terms of the number and types of other professionals participating in the conferences.
- Procedure: Conferences are guided by Jonsen’s four-box model of clinical ethics (Figure 2). Ladder II screening should also be conducted. Screening is performed by nurses, and patients who meet the screening criteria proceed to Ladder III. Nurses are responsible for documenting multidisciplinary conferences and screening outcomes.

### **Family** **conferences**

- Purpose: To confirm patient wishes or surrogate decisions regarding treatment and care goals
- Timing: If, during a multidisciplinary bedside conference, the need for a family meeting is identified, a family meeting should be conducted as soon as possible.
- Participants: Physician and nurse (preferably the primary nurse or another nurse, if unavailable) attendance is mandatory. The presence of a surrogate decision-maker is required unless attendance by the decision-maker is feasible. A patient should participate in the meeting if they are able to make decisions.
- Procedure: Family meetings should follow Steps 1–4. After the meeting, the attending nurse assesses the family's level of understanding and responds to any questions or concerns. Medical staff participating in the meeting should review the content of the preceding multidisciplinary conference and understand the rationale behind the family meeting. If concerns or needs are identified during routine visits or informal conversations with the family, these should also be recorded in the family meeting notes.

If the patient has no relatives, a meeting should proceed to confirm the patient’s wishes in as far as possible. If this is not feasible, the medical team should determine the best course of action for the patient and consult the hospital’s ethics committee, as needed.

### **Explanation to the patient and family**

The attending physician should explain the current medical condition, the treatments being administered, their effects, and future treatment plans. Communication tools recommended by Vital Talk, including the SPIKES protocol (for delivering bad news), NURSE (for addressing emotions), and REMAP (for discussing the goals of care), should be utilized (Ito, 2023).

### **Confirmation of patient values and wishes regarding current treatment options**

The goal of this program is to improve the QODD in the ICU, with reference to how well the dying process and moment of death align with the patient’s wishes. Understanding patient values and providing care consistent with these values contribute to this goal. The following aspects should be confirmed:

- The patient’s core values and personality characteristics
- The patient and family’s understanding of the illness and previous explanations received
- The patient’s wishes or, when unavailable, presumed wishes
- The family's perspectives and concerns

### **Determination of treatment and care goals**

Treatment and care goals should be established by integrating the patient’s wishes and values, the presumed wishes and values interpreted by the family or surrogate decision-makers, and the healthcare team’s professional judgment regarding the most appropriate care.

### **Assessment of family needs**

Any concerns, anxieties, or support needs are identified and the extent to which the family’s needs are met is assessed.

**Examples of family needs include:**

- Timely updates on the patient’s condition, treatment, and prognosis
- Support for understanding the information provided
- Assistance in making surrogate decisions that respect the patient’s wishes
- Flexibility in terms of visitation hours
- Information concerning social support services
- Opportunities to participate in care activities

## **Ladder Ⅲ: EOL care**

- Purpose: to reduce the psychological distress and stress experienced by families during the dying process of a patient for whom life-saving treatment is deemed no longer feasible or who has expressed a wish to withhold further treatment, and to facilitate the grieving process
- Timing of implementation: EOL care is initiated when Ladder II screening at a multidisciplinary bedside conference determines that the patient is unlikely to survive and is approaching death.
- Implementation method: EOL care consists of the provision of information regarding EOL; identification and fulfillment of patient and family needs; symptom relief; and post-bereavement follow-up. Measures from Ladders I and II should continue concurrently.

### **Symptom relief**

Symptom management should continue in accordance with Ladder I: Evidence-based symptom management, even after transitioning to EOL care.

### **Provision of EOL information and confirmation of family understanding**

**Information provided by the physicians**

The attending physician explains to the family that the patient is not responding to treatment and that life-saving measures are no longer feasible. When providing information, the communication strategies proposed by Vital Talk (see supplementary materials) should be employed, including the SPIKES protocol (for delivering bad news), the NURSE framework (for responding to emotions), and the REMAP approach (for goal-of-care discussions) (Ito, 2023).

**Information provided by the nurses**

Nurses should provide the family with information concerning signs or symptoms that a patient may experience as death approaches, and potential psychological responses that the family may experience during bereavement. The NURSE framework can also be used to respond to emotions.

Examples of information provided:

- Changes in breathing: in a non-ventilated patient, breathing may slow, speed up, or pause; mandibular breathing (jaw movements) may also be observed, which is not typically distressing for patients. If signs of dyspnea are observed or reported, ventilator support or sedative dosage can be adjusted.
- Changes in blood pressure and heart rate: blood pressure often decreases, and the heart rate may become irregular or fluctuate. Moreover, extremities may feel cold or appear pale. Warm blankets can be used to provide comfort.
- Changes in consciousness and responsiveness: patients may become agitated, delirious, or comatose. Even in coma, hearing may remain. Gentle touch and communication with family members can provide comfort.
- Agitation: patients may appear restless or disturbed. Nurses will consider and address the possible causes (pain, dyspnea, or anxiety).
- Delirium, common in patients in the ICU who are at a terminal stage, is characterized by confusion, hallucinations, or agitation. Therefore, symptom management and sedation should be considered.
- Treatment environment (physical and structural): arterial lines and other invasive devices may be removed with physician approval if they cause discomfort. If the patient/family desires and the physician agrees, transfer to a private ICU room or general ward may be arranged to ensure a quieter environment.
- Family participation in care: family members may assist with care activities, such as wiping the patient’s face or body or massaging their hands and feet.
- Post-intensive care syndrome-family (PICS-F): Families of patients in the ICU may experience psychological distress, especially after death. Symptoms, such as insomnia, anxiety, and depression may persist for months. Families are encouraged to consult with ICU staff or seek counseling or psychiatric support if symptoms continue.

**Confirmation of family understanding**

- After the physician and/or nurse provides explanations, the family should be asked if they have any questions, and their level of understanding should be confirmed.

### **Identifying and fulfilling the needs of the patient and family in relation to the bereavement**

The care team identifies and fulfills the needs of the patient and their family during the EOL period. With reference to Reeve et al.’s 2021 ‘Three Wishes Project’, the team seeks to elicit and honor three specific personal wishes from the patient and/or family. These wishes typically fall into the following categories: (A) enhancing connections with family and friends, (B) creating a personalized ICU environment, (C) honoring the patient as a unique individual, (D) providing care for the family, and (E) supporting religious or spiritual rituals.

Examples include playing a patient’s favorite music, allowing pets to visit, displaying personal items, facilitating virtual or in-person visits with loved ones, and arranging religious rites.

**Examples of the three wishes:**

| Wish category | Examples |
| --- | --- |
| Enhancing connections with family and friends | - Extend visiting hours and remove visitor limits - Help locate or reconnect with estranged family members - Share memories together |
| Creating a comforting environment | - Watching favorite TV shows - Display photos of meaningful places - Bring personal belongings (e.g., decorations, stuffed animals, music) - Decorate the room for seasonal celebrations - Remove unnecessary lines or catheters - Discontinue physical restraints - Transfer to a private ICU room or general ward if possible |
| Honoring the patient as a unique individual | - Cut or style hair, paint nails - Dress the patient in their own clothing (e.g., hat, pajamas) - Offer favorite foods or drinks (via flavored swabs or feeding tube) - Take the patient for a walk outside the ICU - Respect and follow advance directives |
| Providing care for the family | - Offering a private waiting room - Provide bereavement support - Refer to a psychological counselor |
| Spiritual or religious rituals | - Arrange visits by clergy or religious group members - Organize a farewell ceremony |
| Keepsakes and remembrance | - Gift a lock of hair or a framed handprint - Record and present the patient’s heartbeat as a keepsake - Take family photos at the bedside |

### **Psychosocial support**

**Before death**

- Provide EOL information and confirm the family’s understanding
- Identify and fulfill the needs of the patient and family related to the bereavement
- Create opportunities and a supportive environment for the family to ask questions and express emotions
- Practice active listening and show empathy toward the family’s thoughts and concerns
- Confirm the family’s preferences regarding being present at the time of death
- Assess the need for spiritual services or religious support

**At the time of death**

- Provide EOL information and confirm the family’s understanding
- Identify and fulfill the needs of the patient and family related to the bereavement
- Create opportunities and a supportive environment for the family to ask questions and express emotions
- Practice active listening and show empathy toward the family’s thoughts and concerns
- Adjust the visiting environment: relax restrictions on visiting times and number of visitors, ensure privacy, and allow time alone with the patient if desired

**After death**

- Express condolences and acknowledge the loss
- Create opportunities and a supportive environment for the family to ask questions and express emotions
- Practice active listening and show empathy toward the family’s thoughts and concerns
- Invite questions related to the EOL process and provide sensitive answers

### **Post-bereavement follow-up**

Depending on the institutional system, post-bereavement follow-up may include counseling services or referrals to post-intensive care syndrome (PICS) outpatient clinics.

## **Figure 1.** Quality improvement ladder for palliative care and quality of dying and death in the ICU


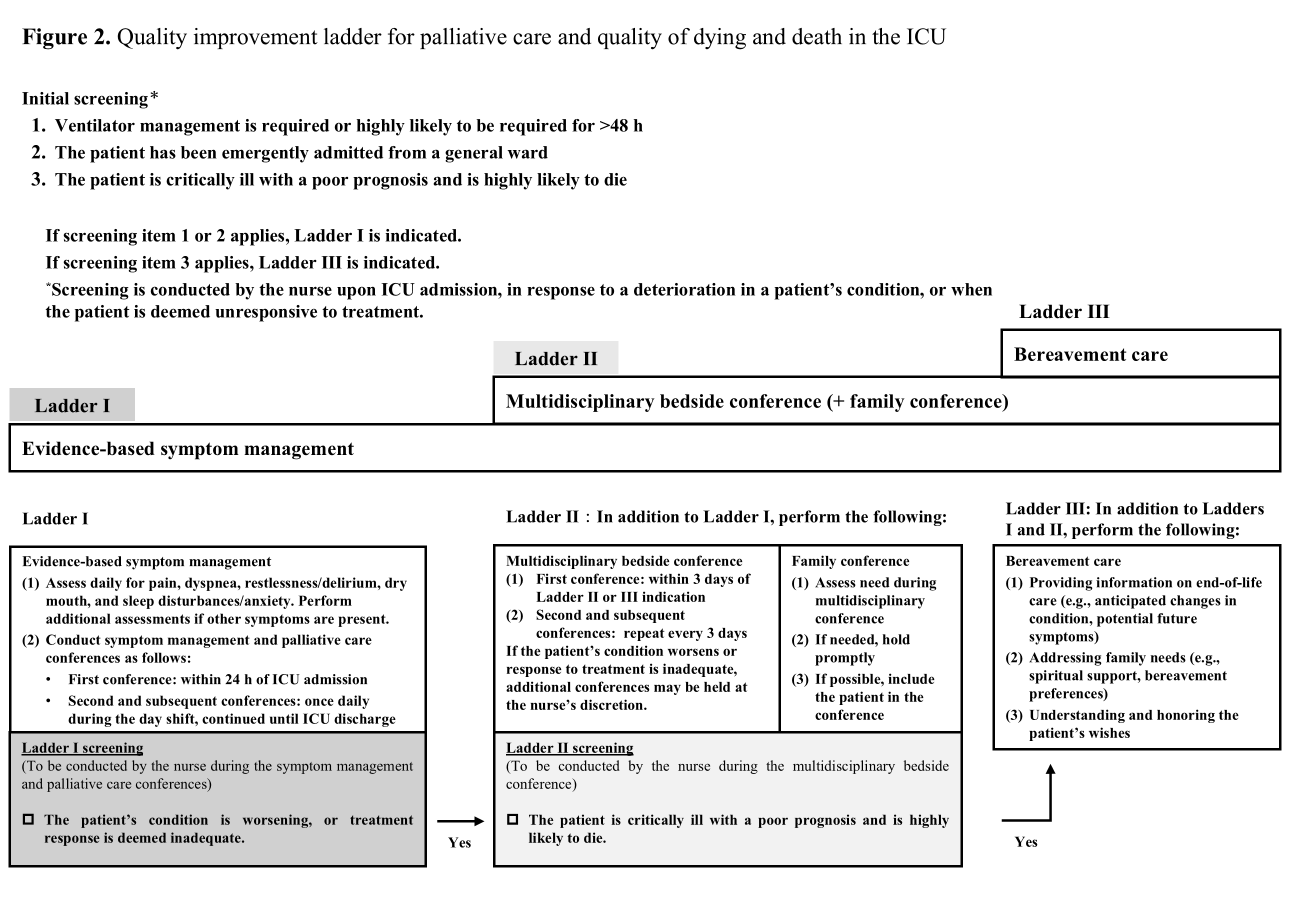


## **Figure 2.** The four topics approach to clinical ethics case analysis


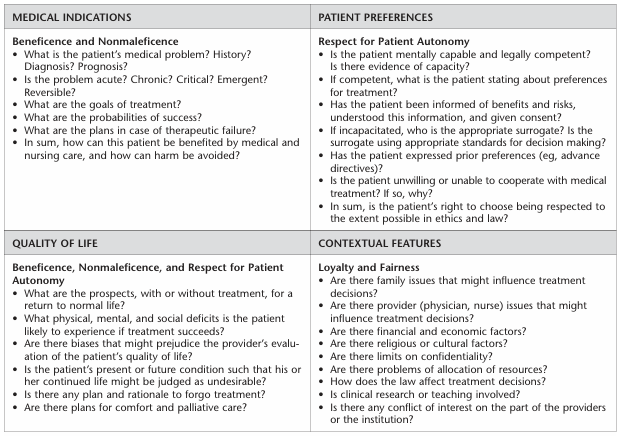


## **References**

Bergeron, N., Dubois, M. J., Dumont, M., Dial, S., & Skrobik, Y. (2001). Intensive Care Delirium Screening Checklist: Evaluation of a new screening tool. Intensive Care Medicine, 27(5), 859–864. https://doi.org/10.1007/s001340100909

Blinderman, C. D., & Billings, J. A. (2015). Comfort care for patients dying in the hospital. The New England Journal of Medicine, 373(26), 2549–2561. https://doi.org/10.1056/NEJMra1411746

Devlin, J. W., & Skrobik, Y. (2018). Clinical practice guidelines for the prevention and management of pain, agitation/sedation, delirium, immobility, and sleep disruption in adult patients in the ICU. (Japanese translation).

Edwards, J. D., Voigt, L. P., & Nelson, J. E. (2017). Ten key points about ICU palliative care. Intensive Care Medicine, 43(1), 83–85. https://doi.org/10.1007/s00134-016-4481-6

Ito, K. (2023). Emergency medical care response. Journal of Medicine and Society, 33(1), 53–66.

Jonsen, A. R., Siegler, M., Winslade, W. J., Akabayashi, A., Kurata, N., & Kodama, S. (2006). Clinical ethics: A practical approach to ethical decisions in clinical medicine (Japanese translation).

Kakinoki, Y., Maki, Y., Ogasawara, T., Koseki, K., Nishihara, T., Kikutani, T., ... Kishimoto, E. (2008). Guideline for Diagnosis of Dry Mouth in the Disabled. Journal of the Japanese Association for Dental Science : JJDS (27), 30-34.

Koga, Y., Murata, H., & Yamase, H. (2014). Validity and reliability of the Japanese version of the CAM-ICU flow sheet. Yamaguchi Medical Journal, 63(2), 93–101.

Kentish-Barnes, N., Chevret, S., Valade, S., Jaber, S., Kerhuel, L., Guisset, O., ... Azoulay, E. (2022). A three-step support strategy for relatives of patients dying in the intensive care unit: A cluster randomised trial. The Lancet, 399(10325), 656–664. https://doi.org/10.1016/S0140-6736(21)02176-0

Murata, H., Oono, Y., Sanui, M., Saito, K., Yamaguchi, Y., Takinami, M., Richards, K. C., & Henker, R. (2019). The Japanese version of the Richards-Campbell Sleep Questionnaire: Reliability and validity assessment. Nursing Open, 6(3), 808–814. https://doi.org/10.1002/nop2.252

Japan Academy of Critical Care Nursing, Committee on Oral Care. (2021). Practical oral care guide for intubated patients. https://jaccn.jp/assets/file/guide/OralCareGuide_202102.pdf

Japan Academy of Critical Care Nursing, Committee on End-of-Life Care. (2019). End-of-life practice guide in emergency and intensive care settings. https://jaen.jp/assets/file/EOL_guide/EOL_guide1.pdf

Payen, J. F., Bru, O., Bosson, J. L., Lagrasta, A., Novel, E., Deschaux, I., Lavagne, P., & Jacquot, C. (2001). Assessing pain in critically ill sedated patients by using a behavioral pain scale. Critical Care Medicine, 29(12), 2258–2264. https://doi.org/10.1097/00003246-200112000-00004

Puntillo, K. A., Arai, S., Cohen, N. H., Gropper, M. A., Neuhaus, J., Paul, S. M., & Miaskowski, C. (2010). Symptoms experienced by intensive care unit patients at high risk of dying. Critical Care Medicine, 38(11), 2155–2160. https://doi.org/10.1097/CCM.0b013e3181f267ee

Puntillo, K., Nelson, J. E., Weissman, D., Curtis, R., Weiss, S., Frontera, J., ... Campbell, M. (2014). Palliative care in the ICU: Relief of pain, dyspnea, and thirst—a report from the IPAL-ICU Advisory Board. Intensive Care Medicine, 40(2), 235–248. https://doi.org/10.1007/s00134-013-3153-z

Reeve, B. K., Dennis, B. B., Dechert, W., Longo, B., Heels-Ansdell, D., Scholes, A., ... Cook, D. J. (2021). Community implementation of the 3 Wishes Project: An observational study of a compassionate end-of-life care initiative for critically ill patients. CMAJ Open, 9(3), E757–E764. https://doi.org/10.9778/cmajo.20200273

Sakuramoto, H., Hatozaki, C., Unoki, T., Aikawa, G., Kobayashi, S., Okamoto, S., ... Fukui, M. (2021). Translation, reliability, and validity of the Japanese version of the Respiratory Distress Observation Scale. PLOS ONE, 16(8), e0255991. https://doi.org/10.1371/journal.pone.0255991

Unoki, T., Sakuramoto, H., Okimura, A., Takeshima, C., Aoki, K., Otani, N., & Yanagisawa, Y. (2010). Development of the Japanese version of the Richmond Agitation-Sedation Scale (RASS). Journal of the Japanese Society of Intensive Care Medicine, 17(1), 73–74.

Yamada, A., & Ikematsu, Y. (2016). Validity, reliability, and responsiveness of the Japanese version of the Critical-Care Pain Observation Tool (CPOT-J). Journal of the Japanese Society of Intensive Care Medicine, 23(2), 133–140.

## **Supplementary material:** Communication guide for patients and families

### **1. SPIKES (Setup / Perception / Invitation / Knowledge / Emotion / Summarize)**

**Setup – Preparing for the conversation**

- Ensure that necessary information (medical facts and a plan), personnel (e.g., nurses, social workers), and an appropriate setting (quiet, private space) are in place
- Turn off mobile phones or delegate urgent responsibilities to another physician in advance

**Perception – assessing the patient’s and family’s understanding**

- Clarify what the patient or family has previously been told and how they understand the current situation. Example: “What have you been told so far about the illness?” or “What is your understanding of the current situation?”

**Invitation – seeking permission to share information**

- While most patients and families want full disclosure regarding the diagnosis, prognosis, and treatment options, some may prefer not to know all the details.
- Avoidance of information can be a coping mechanism, especially in serious illness
- Ask: “Would it be okay if I explain the current situation and test results?” or “Would you prefer a brief overview now and a more detailed discussion about treatment options later?”

**Knowledge – delivering information clearly and simply**

- Avoid medical jargon and use language that is easy to understand.
- When delivering bad news, use a warning statement such as: “I’m afraid I have some serious news to share” or “Unfortunately, this may not be what you were hoping to hear.”
- After delivering the news, a silence of at least 10–15 s is allowed so that the patient and family had time to absorb the information and respond.

**Emotion – responding to emotions**

- Use empathetic statements to acknowledge and respond to emotional reactions.
- Use the NURSE framework (explained below) to express empathy.
- Avoid trying to immediately ‘fix’ the situation by offering treatments that may be unrealistic. Instead, invite further questions and address any concerns.

**Summarize – summarizing the conversation and planning next steps**

- Summarize the information shared and outline the next steps (e.g., additional tests and treatment decisions)
- Ask: “Does this plan sound right for you?” or “Is the next step clear to you?” to confirm understanding

### **2. NURSE (Naming/Understanding/Respecting/ Supporting/Exploring)**

**Naming – naming the emotion**

- Name or paraphrase the emotions expressed by the patient or family to show empathy.

Examples:

“You seem very worried about your loved one’s condition.”

“This news must have come as a shock.”

“It sounds like you’re concerned about ___.”

**Understanding – expressing understanding**

- Acknowledge and validate emotions

Example: “I understand that you’re feeling anxious about ___.”

Or “I can only imagine how overwhelming this must be.”

**Respecting – showing respect**

- Use verbal or non-verbal responses to affirm the family’s or patient’s experiences

Example: “You’ve done so much to support your loved one through this.”

A nod, warm tone, or gentle touch can also express respect.

**Supporting – offering support**

- Clearly express your ongoing support

Example: “We are here for you and will do everything we can to help.”

“Please feel free to talk to us anytime.”

**Exploring – exploring further**

- Further investigate emotional statements to better understand their meaning

Example: If someone says, “Why is this happening?” respond with:

“What are you most worried about right now?”

This can help uncover core values or concerns, which may guide further decision-making.

### **3. REMAP (Reframe / Expect / Map / Align / Plan)**

**Reframe – communicate a change in the clinical situation**

- When current treatments are no longer effective, clinicians must reassess treatment goals. In this first step, the clinician evaluates how well the patient and family understand disease progression and provides new information if needed.

Examples:

| Physician | How do you perceive the current situation? |
| --- | --- |
| Patient or family | It doesn’t feel like things are improving. They seem to be in more pain than before. |
| Physician | Would it be alright if I spoke in more detail about the current condition? |
| Patient or family | Yes, I’d like to understand more. |
| Physician | We have done everything we can in terms of treatment, but we think it may be time to reassess the situation. At this point, continuing further treatment might bring more harm than benefit to the patient (to you). |
| Physician | We will do everything we can that aligns with your wishes—or what we understand to be the patient's presumed wishes. But I also want to ask you to consider that things may not go the way you hope. |

- Start by asking what the patient and family already know, and wait for their responses. If they already understand the seriousness of the condition, simply confirming their understanding may suffice as a reframe. However, if they are unaware of clinical reality, reframing will involve delivering significant or difficult news.

**Expect emotion – respond to emotional reactions**

- Emotional responses from patients and their families after reframing may be verbal or nonverbal, such as crying. These responses indicate that bad news was received.
- Address these emotions using empathic communication techniques (also see ‘2. NURSE’).

**Map out patient values – explore what matters most**

- Before revising the treatment plan, clinicians should initiate value-based conversations by saying, for example:

“To help us plan what is best for you right now, I would like to take a few minutes to understand what matters most to you.”

- If direct communication with the patient is not possible, ask the family to explore the patient’s presumed wishes and values.

Examples:

| When communication with the patient is possible | What is your greatest concern after hearing about your current condition? |
| --- | --- |
|  | What is the most important thing to you as we move forward with treatment? |
| When communication with the patient is difficult (ask the family) | What kind of person was [patient's name]? |
|  | How do you think [patient's name] would make decisions about their treatment? |

**Align with values – ensure treatment goals reflect the patient’s values**

- Once key values have been identified, align treatment options accordingly.
- Summarize what was shared and confirm:

“From what you’ve shared, it sounds like [you/your father/your loved one] feels that ___ is most important.”

- After confirming mutual understanding, move on to discuss treatment options.

**Propose a plan – recommend a medically appropriate and value-concordant plan**

- Based on a patient’s values (or presumed wishes), a treatment plan that is both medically appropriate and realistically achievable is proposed.
- Treatment options typically fall into one of three categories: (A) continuous curative treatment, (B) a time-limited trial of curative treatment and, if ineffective, a shift to comfort-focused care, and (C) a focus solely on symptom relief and comfort care, forgoing curative treatment.

**Complete the process**

- If there is agreement on the proposed plan, ask:

“Do you have any questions about what we discussed?”

- Encourage exploration of all aspects of the treatment plan and ensure clarity.

**References**

Back, A. L., Arnold, R. M., Baile, W. F., Tulsky, J. A., & Fryer-Edwards, K. (2005). Approaching difficult communication tasks in oncology. CA: A Cancer Journal for Clinicians, 55(3), 164–177. https://doi.org/10.3322/canjclin.55.3.164

Childers, J. W., Back, A. L., Tulsky, J. A., & Arnold, R. M. (2017). REMAP: A framework for goals of care conversations. Journal of Oncology Practice, 13(10), e844–e850. https://doi.org/10.1200/JOP.2016.018796

Baile, W. F., Buckman, R., Lenzi, R., Glober, G., Beale, E. A., & Kudelka, A. P. (2000). SPIKES—A six-step protocol for delivering bad news: Application to the patient with cancer. The Oncologist, 5(4), 302–311. https://doi.org/10.1634/theoncologist.5-4-302

VitalTalk. (n.d.). Quick guides. https://www.vitaltalk.org/resources/quick-guides/
