# Supplementary material for: Development and external validity of a nurse-led intervention program to improve palliative care and quality of dying and death in intensive care unit
Source: PLoS One. 2026 Apr 10;21(4):e0346585. doi: 10.1371/journal.pone.0346585 (PMC13068268; doi:10.1371/journal.pone.0346585)
Supplement: S4 Text — (DOCX) [file pone.0346585.s004.docx]

**S4 Text. Screening and conference items**

| ◆ Primary screening (conducted by nurses upon ICU admission, when a patient’s condition worsens, or when the response to treatment is deemed poor) | |
| --- | --- |
| 1. The patient requires or is highly likely to require mechanical ventilation for >48 h | □ |
| 1. Emergency admission from a general ward | □ |
| 1. Survival is unlikely, and the risk of death is extremely high | □ |
| □ If item 1 or 2 applies, implement Ladder I  □ If item 3 applies, implement Ladder III | |

| ◆ Basic information related to the quality of dying and death (to be recorded at the time of primary screening) | |
| --- | --- |
| [Family information] | Surrogate decision-maker＊ □ Yes ・ □ No  Relationship: (　　　　) |
| [Patient information] | The patient is a non-native Japanese speaker: □ Yes ・ □ No |
| [Patient preferences] | Advance directive (verbal or written): □ Yes ・ □ No |
|  | If an advance directive exists, confirm the following:  □ Implementation of cardiopulmonary resuscitation  □ Use of mechanical ventilation for life-prolonging purposes  □ Use of other life-prolonging mechanical support (e.g., extracorporeal circulation, dialysis) |
|  | Willingness to donate organs: □ Yes ・ □ No |
| *The surrogate decision-maker may not be a single individual; decisions may involve multiple people or an entire family | |

| ◆ Symptom management / palliative care conference (conducted daily after initiation of a ladder) |
| --- |
| 1. Current symptoms (based on a patient’s subjective evaluation or healthcare provider’s objective assessment): |
| □ Pain ・ □ Dyspnea ・ □ Agitation/delirium ・ □ Dry mouth/oral dryness  □ Sleep disturbance ・ □ Anxiety ・ □ Other: (　　　　　　　　　　)  *If symptoms have not been evaluated, conduct the conference again after assessment. |
| 1. Management status of symptoms identified in item 1: |
| □ Symptoms are evaluated and recorded using appropriate methods  □ Interventions have been implemented, and symptoms are either alleviated or have not worsened  *If neither box is checked, review the methods of symptom relief. |
| 3. Methods of intervention for symptom relief (including additions or modifications to standard care): |
|  |
| 4. Necessity of consultation with the palliative care team: |
|  |
| Ladder I screening |
| □ The patient’s condition is worsening or their response to treatment is deemed to be poor  → If applicable, proceed to Ladder II |

| ◆Interdisciplinary bedside conference (conducted within 3 days after starting Ladder II, then repeated every 3 days) |
| --- |
| [Date of conference] |
| Year　　Month　　Day (　　),　　　From: 　　:　　 To: 　　: |
| [Names and professions of participants] |
|  |
| 1. Medical indications |
| 1. Probable clinical trajectory: 2. Goals of treatment and care: 3. Treatment options (that can be presented to the patient/family): |
| 1. Patient preferences |
|  |
| 1. Quality of life |
|  |
| 1. Contextual features |
|  |
| 1. Need for a family meeting |
| □ Yes ・ □ No |
| Ladder II screening |
| □ Survival is unlikely, and the risk of death is extremely high  → If either condition applies, proceed to Ladder III |
| - The conference is guided by the four-box method of clinical ethics - Each item is discussed to determine what specific future actions can be taken |

| ◆ Family meeting |
| --- |
| [Date of meeting] |
| Year　　Month　　Day (　　),　　　From: 　　:　　 To: 　　: |
| [Names and professions of participants] |
|  |
| [Family members present (relationship to patient)] |
|  |
| 1. Information provided to the patient/family |
|  |
| 2. Confirmation of the patient’s values and their known or presumed wishes regarding current treatment options |
| 1. The patient's values and personality: 2. Understanding and interpretation by the patient/family (confirm what explanations have been provided to date): 3. The patient’s wishes or presumed wishes: 4. The family's thoughts: |
| 3. Determination of treatment and care goals |
|  |
| 4. The family’s needs |
|  |
| - If an interdisciplinary bedside conference determines that a family meeting is necessary, it should be held as early as possible - If a patient is capable of making decisions, they should participate in the discussion - Attendance of the surrogate decision-maker is required for family meetings - In situations where a patient has no relatives, a meeting should be held to confirm the patient's wishes in as far as possible. If confirming a patient’s wishes is difficult, the healthcare team should discuss the best possible options for the patient, and consult the hospital's ethics committee if necessary. - Even outside of formal family meetings, if a family’s thoughts, concerns, or needs are expressed during visits or conversations, these should be documented in the family meeting record. |
