# Supplementary material for: Development and external validity of a nurse-led intervention program to improve palliative care and quality of dying and death in intensive care unit
Source: PLoS One. 2026 Apr 10;21(4):e0346585. doi: 10.1371/journal.pone.0346585 (PMC13068268; doi:10.1371/journal.pone.0346585)
Supplement: Attachment — Submitted filename: Response_to_Reviewers.docx [file pone.0346585.s006.docx]

Dear Dr. JONATHAN BAYUO

Thank you the opportunity to revise and resubmit our manuscript, “**Development and external validity of a nurse-led intervention program to improve palliative care and quality of dying and death in intensive care unit**,” to *PLOS One*. We sincerely appreciate the editor’s handling of our submission and the reviewers’ careful and constructive evaluations, which have been invaluable in strengthening the manuscript.

In response to the reviewers’ comments, we have revised the manuscript accordingly and provide a detailed, point-by-point response below outlining how each concern was addressed. We believe that these revisions have substantially improved the clarity, rigor, and transparency of the work, and we respectfully resubmit the manuscript for your further consideration.

**Reviewer #1:**

***Comment 1. Abstract: please give a short background of the study and use paragraph to make it more organized.***

**Response:**

To address this, we have revised the Abstract to include a brief background of the study and reorganized it into clearly defined paragraphs corresponding to the Introduction, Methods, Results, and Conclusion sections to improve clarity and readability.

***Comment 2. Introduction: You have provided the background of your study and identified the gap in previous studies in the similar field***

**Response:**

We appreciate the reviewer’s recognition that the introduction adequately provides the background of the study and clearly identifies the research gaps in previous studies.

***Comment 3. Methods: Did you yourself conducted both the scoping review, and systematic review and meta analysis?***

**Response:**

Yes, we conducted the scoping review, systematic review, and meta-analysis; however, this was not clearly stated in the original manuscript. We have revised the Methods section to clarify this point and added relevant references.

Page 4, line 87-91: Initially, we conducted a systematic review and meta-analysis (SR/MA) [3] and a scoping review (ScR) [7]. The findings from these reviews were synthesized and applied in Steps 1–3. Step 4 involved drafting a preliminary version of the intervention program and assessing its internal and external validity. Based on the results, the program was revised and finalized.

***Comment 4. Step 4, Can you explain the intervention program in detail?***

**Response:**

To address the comment, in the revised manuscript, we have provided a detailed description of the intervention program, including both the original version and revisions made based on expert feedback. Initially, the program was designed as a time- and condition-based flow, reflecting the clinical trajectories of critically ill patients in the ICU. Following expert feedback, we reorganized the intervention into a stepwise ladder-like format to clarify the operational criteria for transitions between levels and to provide a more structured guide for clinical practice. We have added a description of the original flow-based program, the rationale for the reorganization, and details of each ladder level (I–III) to the Methods section. This allows readers to understand both the original program and the modifications made to enhance clarity, feasibility, and applicability in the ICU setting.

Page 11, line 205-222: Based on these eight intervention strategies, we developed a preliminary nurse-led intervention program to improve palliative care and QODD in ICU. Rather than following a ladder-based approach, the intervention was designed with a time- and condition-dependent flow of care. Patients were screened based on their clinical status over time and subsequent steps in the intervention proceeded according to this screening, creating a structured flow throughout the ICU stay. A flow diagram was developed to visually guide the practice.

Given the complexity of transitioning from curative to EOL care in modern ICUs, it is necessary to identify appropriate patients at an early stage, rather than focusing only on those who are near death. Therefore, primary screening criteria were established to identify eligible patients from among all ICU admissions. Once identified, evidence-based symptom management was initiated on ICU admission. For these patients, multidisciplinary bedside conferences were held within 24 h to clarify the goals of care and discuss treatment strategies. This step enabled the early planning of family conferences and timely initiation of EOL care. Nurses closely involved in daily patient care serve as key agents of the program. Their roles in symptom assessment, patient identification, and coordination of multidisciplinary meetings were explicitly defined and formally integrated into the intervention procedures. The intervention program was compiled into a booklet including an intervention flow diagram and designed for distribution to the departments participating in the intervention.

Page 20-21, line 272-287: The primary screening items were revised in response to these comments, and the intervention process, which originally followed a time- and condition-based flow, was reorganized into a stepwise ladder-like format (Fig. 2). This allowed for the initiation of symptom management from the time of ICU admission without altering the overall target population specified in the intervention program.

Following this reorganization, explicit operational criteria were defined for transitions between ladder levels (Fig. 2). Ladder I was initiated for patients who required, or were highly likely to require, mechanical ventilation for more than 48 hours, or who were admitted emergently from a general ward. The transition from Ladder I to Ladder II was considered when the patient’s condition deteriorated or the response to treatment was judged to be poor. Nurses conducted this assessment during daily symptom management conferences. The transition from Ladder II to Ladder III was determined when life-saving treatment was judged difficult and the likelihood of death was extremely high. This decision was made by the nurses during multidisciplinary bedside conferences. In cases of rapid clinical deterioration or an extremely poor response to treatment, a direct transition from Ladder I to Ladder III was permitted. All screening and transition decisions were nurse-led and reassessed based on changes in the patient’s clinical condition.

***Comment 5. Line 119: why did you exclude the participant who had experienced the loss of a family member within the past 6 months?***

**Response:**

Individuals within six months of bereavement were excluded because early grief reactions and psychological distress are generally the most pronounced during this period. Because this intervention program focuses on improving the quality of dying and death, participation can evoke memories of recent losses and potentially exacerbate psychological distress. Therefore, to minimize the emotional burden and ethical risk to the participants, individuals who had experienced the loss of a family member within the past six months were excluded.

Page 6, line 133-137: (g) participants who had experienced the loss of a family member within the past six months. Since early bereavement reactions and psychological symptoms tend to be most pronounced within the first six months following loss [11], these individuals were excluded to minimize psychological distress and avoid triggering recollection of recent bereavement.

***Comment 6. Results: Great! you have clearly explained the details you undertook in each step. Discussion: well written***

**Response:**

Thank you very much for your positive and encouraging comments. They were very encouraging and motivating for our research team.

***Comment 7. Conclusion: Please restate the main aspects of the intervention program***

**Response:**

To address this, we have revised the conclusion to clearly restate the main aspects of the intervention program. Specifically, we have added a description that the program consists of symptom management as its core component, along with three additional key elements.

Page 29, line 445-452: We developed a nurse-led intervention program to improve palliative care and QODD in ICUs following the 6SQuID framework. The program centers on symptom management and includes three additional core components: multidisciplinary bedside conferences, family conferences, and structured end-of-life care. The content and external validity of the program were established through a literature review and input from an expert panel. Therefore, this program has potential utility in enhancing the quality of palliative and EOL care in Japanese ICUs. Furthermore, by adhering to a scientifically rigorous developmental process, this approach offers a model for adapting evidence-based interventions to diverse cultural and healthcare contexts beyond Japan.

**Reviewer #2:**

***Comment 1. Clarity in the transition between ladder levels: Although adjusted based on expert feedback, it would be helpful to explain in more detail the operational criteria for moving between levels, especially between Ladder II and Ladder III.***

**Response:**

We have added detailed explanations regarding the operational criteria for transitions between ladder levels in the Results section. Specifically, we describe the clinical conditions and decision criteria for transitions from Ladder I to II, from Ladder II to III, and from Ladder I directly to III. In addition, we clarified who performed the assessment and at what times. These explanations are consistent with the information presented in Figure 2.

Page 20-21, line 277-287: Following this reorganization, explicit operational criteria were defined for transitions between ladder levels (Fig. 2). Ladder I was initiated for patients who required, or were highly likely to require, mechanical ventilation for more than 48 hours, or who were admitted emergently from a general ward. The transition from Ladder I to Ladder II was considered when the patient’s condition deteriorated or the response to treatment was judged to be poor. Nurses conducted this assessment during daily symptom management conferences. The transition from Ladder II to Ladder III was determined when life-saving treatment was judged difficult and the likelihood of death was extremely high. This decision was made by the nurses during multidisciplinary bedside conferences. In cases of rapid clinical deterioration or an extremely poor response to treatment, a direct transition from Ladder I to Ladder III was permitted. All screening and transition decisions were nurse-led and reassessed based on changes in the patient’s clinical condition.

***Comment 2. Stronger justification for the inclusion of non-terminal patients: While explained in the discussion, reinforcing this point in the introduction would help avoid misinterpretations about the scope of the program.***

**Response:**

To address this, we have reinforced the rationale for including non-terminal patients in the intervention program by adding an explanation to the background section to clarify the scope of the program.

Page 3, line 63-72: In the ICU setting, patient prognosis is often uncertain at admission and may change over time. Many patients who die in the ICU initially receive aggressive, life-sustaining treatments with the expectation of recovery. Therefore, discussions regarding patient values, preferences, and advance directives should be initiated early after ICU admission and revisited as clinical conditions evolve. Inadequate symptom control is a major contributor to poor QODD [4], and ICU patients frequently experience significant unrelieved physical and psychological distress [5]. Accordingly, palliative care should be provided to all ICU patients regardless of their prognosis. Although ICU palliative care initially focused on EOL care, it is now understood to encompass symptom management, shared decision-making, and support for patients and their families throughout the course of the critical illness [6].

***Comment 3. Describe how qualitative saturation was managed in the FGI phase, given that six experts participated. This is not essential but would increase methodological transparency.***

**Response:**

Although qualitative saturation was not a predefined endpoint for the FGI, it was considered achieved when no new themes or suggestions relevant to refining the intervention program emerged during the later stages of the discussion. The six experts had diverse professional backgrounds relevant to ICU palliative and end-of-life care and their feedback converged on similar key issues, suggesting sufficient information redundancy. In addition, we verified the analysis results with the participants to ensure that their statements were accurately reflected and that no additional opinions were omitted. We have clarified this in the manuscript:

Page 6, line 138-147: Interviews were conducted remotely via video conferencing. Audio recordings were transcribed verbatim and reviewed multiple times. Common themes were identified and synthesized. Revisions of the preliminary program were based on expert feedback. Qualitative saturation was achieved when no new themes or suggestions relevant to the refinement of the intervention program emerged during the later stages of the FGI. To ensure trustworthiness of the content analysis, the data were reviewed repeatedly by two researchers and supervised by a qualitative research expert. The raw data were re-examined to ensure accuracy. Additionally, the analyzed data were re-examined and verified with the participants to ensure that their statements were accurately reflected and that no additional opinions were omitted. The revised version was defined as the “modified intervention program.” The interview guidelines are presented in Table 1.

***Comment 4. English fluency: Some paragraphs could benefit from grammatical simplification, especially in the Methods section. This does not affect comprehension but could increase clarity for international audiences.***

**Response:**

Thank you for your comments. We have revised the manuscript for clarity and readability, particularly in the Methods section. The manuscript has been professionally edited by Editage to ensure grammatical accuracy and improve clarity for an international audience.

***Comment 5. Overall Review Conclusion: The manuscript is robust, rigorous, and provides a relevant intervention program for critical care settings, with the potential to be replicated in other cultural contexts. I recommend publishing it after addressing the minor formatting issues.***

Thank you for your positive evaluation and the constructive feedback. We are encouraged to find the manuscript robust and the intervention program relevant and potentially generalizable. We have addressed the minor formatting issues, as suggested. We look forward to hearing from you and would be happy to make further changes, if required.
