# Supplementary material for: Development and external validity of a nurse-led intervention program to improve palliative care and quality of dying and death in intensive care unit
Source: PLoS One. 2026 Apr 10;21(4):e0346585. doi: 10.1371/journal.pone.0346585 (PMC13068268; doi:10.1371/journal.pone.0346585)
Supplement: Attachment — Submitted filename: Response_to_Reviewers_auresp_2.docx [file pone.0346585.s007.docx]

Dear Dr. JONATHAN BAYUO

Thank you the opportunity to revise and resubmit our manuscript, “**Development and external validity of a nurse-led intervention program to improve palliative care and quality of dying and death in intensive care unit**,” to *PLOS One*. We sincerely appreciate the editor’s handling of our submission and the reviewers’ careful and constructive evaluations, which have been invaluable in strengthening the manuscript.

In response to the reviewers’ comments, we have revised the manuscript accordingly and provide a detailed, point-by-point response below outlining how each concern was addressed. We believe that these revisions have substantially improved the clarity, rigor, and transparency of the work, and we respectfully resubmit the manuscript for your further consideration.

**Reviewer #2:**

***Comment 1. Support for conclusions: The data support the development and refinement of a program with content validity according to experts (CVI), but the abstract statement can be interpreted as a claim of already demonstrated effectiveness/consistency (“enables a more consistent delivery… regardless of individual providers’ knowledge or attitudes”). This exceeds what can be concluded without an implementation/effectiveness evaluation.***

**Response:**

To address this, we have revised the wording in the Abstract to avoid implying demonstrated effectiveness and to better align the conclusions with the scope of this development and content validation study. Specifically, following the reviewer’s suggestion, we replaced “enables a more consistent delivery of end-of-life care in the ICU” with “was designed to facilitate more consistent delivery of end-of-life care in ICU.”

For consistency, we also revised similar wording in the Discussion section.

Page 27, line 399-401: Symptom management in the ICU remains suboptimal. An intervention program designed to facilitate consistent care delivery, independent of the providers’ knowledge or attitudes, may enhance symptom management.

***Comment 2. In the editorial form, they state: “All relevant data are within the manuscript and its Supporting Information files.”***

***But for a study with FGI + CVI, “underlying data” would typically include at least: a matrix/sheet of CVI ratings per item (or complete table), an instrument (survey, interview guide), and, if transcripts cannot be shared, some level of auditable qualitative evidence (e.g., a table of topics with anonymized extracts or a codebook).***

***In the material I reviewed, I can confirm that the statement is there, but I cannot confirm that these inputs are actually accessible as complete underlying data based solely on the system phrase.***

**Response:**

Thank you for your important comment regarding data availability and transparency.

In response, we have ensured that all relevant underlying data are accessible through the Supporting Information files. Specifically, we have added:

S1 Text: Survey instrument used for content validity index (CVI) evaluation

S2 Text: Item-level CVI ratings for each expert

Regarding the FGI data, full interview transcripts cannot be publicly shared due to ethical restrictions and the potential identifiability of participants. However, we have provided a structured summary of themes and representative anonymized excerpts within the manuscript to ensure auditability while protecting confidentiality.

We believe these additions ensure that all relevant underlying data supporting the study findings are now fully accessible in accordance with the journal’s data availability policy.

***Comment 3. As a minor detail, traces of editing are still visible (e.g., small typos/duplications in some lines of the Methods section), but these do not impede comprehension.***

**Response:**

Thank you for this comment. We carefully reviewed the Methods section and corrected minor typographical errors, punctuation issues, and wording duplications throughout the manuscript.
